# Soil-Transmitted Helminth Infections among Plantation Sector Schoolchildren in Sri Lanka: Prevalence after Ten Years of Preventive Chemotherapy

**DOI:** 10.1371/journal.pntd.0001341

**Published:** 2011-09-27

**Authors:** Kithsiri Gunawardena, Balachandran Kumarendran, Roshini Ebenezer, Muditha Sanjeewa Gunasingha, Arunasalam Pathmeswaran, Nilanthi de Silva

**Affiliations:** 1 Department of Parasitology, Faculty of Medicine, University of Kelaniya, Ragama, Sri Lanka; 2 Department of Public Health, Faculty of Medicine, University of Kelaniya, Ragama, Sri Lanka; 3 Department of Infectious Disease Epidemiology, Faculty of Medicine, Partnership for Child Development, Imperial College London, St Mary's Campus, London, United Kingdom; London School of Hygiene and Tropical Medicine, United Kingdom

## Abstract

**Background:**

The plantation sector in Sri Lanka lags behind the rest of the country in terms of living conditions and health. In 1992, a sector-wide survey of children aged 3–12 years and women of reproductive age showed >90% prevalence of soil-transmitted helminth infections. Biannual mass de-worming targeting children aged 3–18 years started in 1994 and was continued until 2005. The present study was carried out to assess the status of infection four years after cessation of mass de-worming.

**Methods/Findings:**

A school-based cross-sectional survey was carried out. Faecal samples from approximately 20 children from each of 114 schools in five districts were examined using the modified Kato-Katz technique. Data regarding the school, the child's family and household sanitation were recorded after inspection of schools and households. Multivariate analysis was carried out using logistic regression, to identify risk factors for infection. Faecal samples were obtained from 1890 children. In 4/5 districts, >20% were infected with one or more helminth species. Overall combined prevalence was 29.0%; 11.6% had infections of moderate-heavy intensity. The commonest infection was *Ascaris lumbricoides*, present in all five districts, as was *Trichuris trichiura*. Hookworm was not detected in two districts. Multivariate analysis identified low altitude and maternal under-education as risk factors for all three infections. Poor household sanitation was identified as a risk factor for *A. lumbricoides* and hookworm, but not *T. trichiura* infections.

**Conclusions/Significance:**

The results indicate that regular mass de-worming of plantation sector children should be resumed along with more emphasis on better sanitation and health education. They show that even after 10 years of mass chemotherapy, prevalence can bounce back after cessation of preventive chemotherapy, if the initial force of transmission is strong and other long-term control measures are not concomitantly implemented.

## Author Summary

Mass de-worming of pre-school and school-age children was introduced in Sri Lanka's plantation sector in 1994 after a survey showed that >90% of children and women of reproductive age were infected with intestinal worms. The present study was carried out to assess the status of infection four years after mass de-worming was stopped in 2005 due to lack of funds. Approximately 20 children from each of 114 schools in five districts were examined. Data regarding the school, the child's family and household sanitation were recorded. Faecal samples from 1890 children were examined for worm eggs. In 4/5 districts, >20% were found infected with one or more intestinal worm. Overall, 29.0% of children were infected and 11.6% of these had moderate–heavy worm burdens. The commonest infection was roundworm, followed by whipworm. Hookworm was not detected in two districts. Statistical analysis identified low altitude, maternal under-education and poor household sanitation as risk factors for infection. The results indicate that when initial infection rates are very high, in the absence of marked improvements in sanitation and health education, even 10 years of biannual mass de-worming may not be enough to prevent resurgence of infection after cessation of mass de-worming.

## Introduction

Sri Lanka has already achieved several Millennium Development Goals such as universal primary school enrolment, gender parity in school enrolment, and is on track to achieve the desired reduction in under-five and infant mortality. Poverty has markedly reduced in the urban and rural sectors between 1990 and 2006, from 26.1% to 15.2% [1]. However, not all parts of the country have benefited equally from these gains. In the plantation sector, which has a resident population of about 939,000 living and working on tea and rubber plantations, there is widespread child malnutrition, maternal mortality rates are exceptionally high and poverty has increased by over 50% in the same period [1].

Soil-transmitted helminth (STH) infections are well-known accompaniments of poverty in the developing world [2]. A survey that covered the entire plantation sector in Sri Lanka in 1992 found over 90% of children to be infected [3]. A major de-worming program, offering bi-annual treatment with 500 mg mebendazole to children aged 3–18 years, was launched in 1994 [4]. However, this programme was discontinued after about ten years, due to lack of funds, without proper reassessment of the epidemiological situation.

A national survey of the health of school children, carried out in 2003, found only 6.9% to be infected with any of the three major STH infections [5]. This is well below the threshold of 20% prevalence recommended by the WHO for implementation of mass de-worming of school children in endemic areas [6]. Despite ten years of mass de-worming between 1994 and 2005 in the plantation sector, the extremely high prevalence of STH infection at the outset of the de-worming programme, and the actual increase in poverty in the intervening period, suggested that the results of the national survey should not be extrapolated to this sector.

This study was designed to estimate the current prevalence and intensity of STH infections among primary school children in the plantation sector and to describe the factors associated with infection, in order to provide data for rational design and targeting of school-based health and nutrition programmes.

## Methods

### Ethics statement

Approval was obtained from the Ethics Review Committee of the Faculty of Medicine, University of Kelaniya (application no P103/08/2009). After selection of children in each school, their homes were visited and their parent(s) interviewed in order to obtain written, informed consent for the child's participation in the study, and to obtain information on the socio-economic status of the family. Only children whose parents gave consent were included in the study. All children were offered treatment with mebendazole 500 mg at the end of the study.

### Study design and setting

This was a school-based, cross-sectional survey. The 2007 School Census of the Ministry of Education identifies 830 ‘plantation sector schools’, almost all of which provide instruction in the Tamil language (also referred to as ‘Tamil-medium schools’). These schools are divided into two categories: inside or outside a plantation. Tamil-medium schools located inside the plantations formed the setting of the study.

### Study area

**Figure 1:**
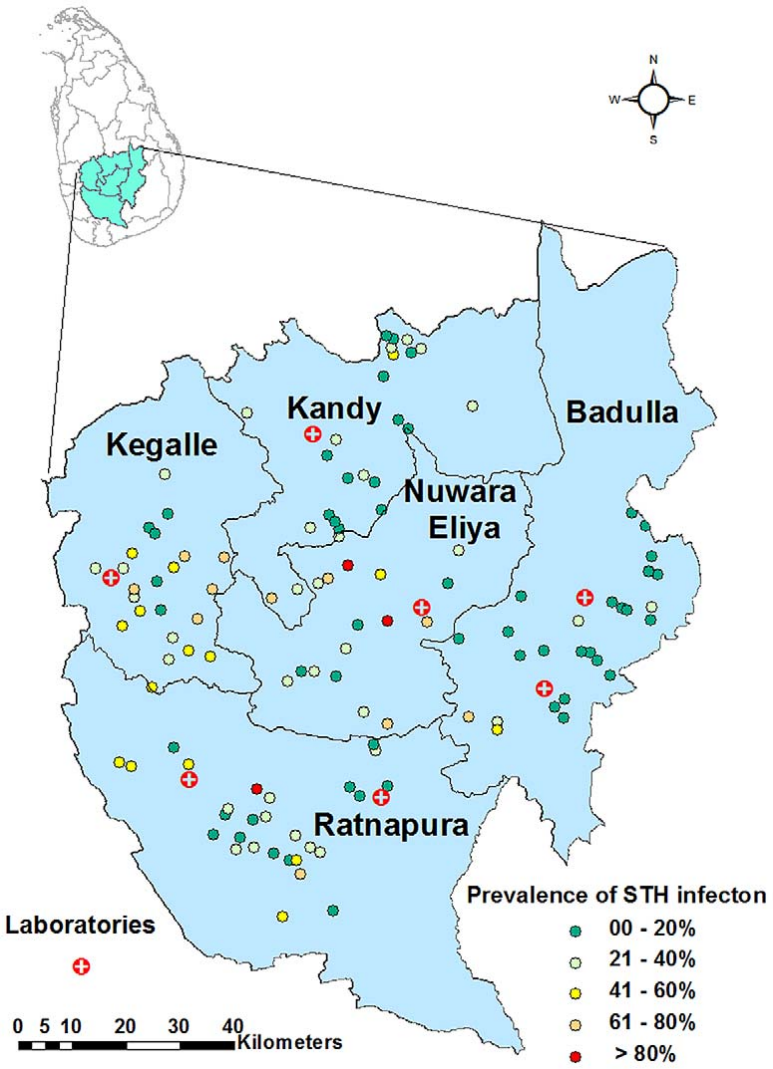
Geographical location of study schools and laboratories in the districts of Kandy, Kegalle, Nuwara Eliya, Badulla and Ratnapura, together with prevalence of infection with any one or more soil-transmitted helminth infection at each school.

The study was performed in five administrative districts, namely Nuwara Eliya, Badulla, Kegalle, Ratnapura and Kandy. These five districts are centrally located in the southern half of Sri Lanka (Figure 1). The study region occupies a total land area of 11,500 km^2^ representing 17.5% of the total land area of Sri Lanka. The 2010 estimated midyear population indicates the region's population as about 3.5 million people, representing 19.1% of the country's population.

### Study population

The study population was selected from among students who were registered in grade 4 classes in 2009, in schools lying within plantations in the five selected districts. Grade 4 classes were selected because this study was part of another larger study which assessed children's cognitive abilities with a test specifically designed for this grade. The schools within this study area include nearly 90% of the primary school population in plantation schools. Schools with less than 60 students were excluded since it was unlikely that they would have the 20 or 27 students in Grade 4 needed for a cluster. Schools with more than 400 students were also excluded since they draw students from a wide geographical area and different society other than from the plantation sector. The eligible schools were considered as clusters for sampling.

### Sample size

Sampling was carried out based on cluster sampling procedure [7]. This involved two stages: firstly, to select the primary units (i.e. schools) within each district, and secondly, to select the elementary sampling units (classes) within the primary units. In Nuwara Eliya district, the sample size calculation was based on the need to determine if the overall prevalence of infection was over the 20% threshold recommended by the WHO for introduction of mass deworming, and the need to ensure that an actual prevalence of 15% or less would not be misclassified as 20%. On this basis, the required sample size for the district was 196; with an anticipated response rate of 70%, this increased to 280. As cluster sampling was to be used, a design effect of 1.5 was also taken into consideration, thus giving a final sample size of 420. For logistical reasons, it was decided that the sample of 420 children would be drawn from16 schools from among 297 schools in the district. In each selected school, 27 children were selected using the lottery method. If a school had less than 27 children in Grade 4, the remainder was made up by selection of children in other grades.

In the other four districts, children included in the study were part of another larger study on the impact of school-based de-worming and iron supplementation on the cognitive abilities of school children. Thus, while the desired minimum sample size per district remained the same as for Nuwara Eliya, the size of a cluster was limited to 20 children per school because of constraints with regard to assessment of cognitive ability. An overall sample size of 100 schools with 20 children per school was selected; if the school had less than 20 children in Grade 4, all children registered in the grade were selected, but numbers were not made up from other classes.

Data collection in the field was conducted in July 2009 in Nuwara Eliya District; and between September and November 2009 in the other four districts.

### Parasitological assessment

Faecal examination for helminth infections was carried out by experienced medical laboratory technicians. A single smear per faecal sample was examined using the modified Kato-Katz technique as recommended by WHO [8]. Samples were left to clear for 20–60 minutes before reading. Kits for the Kato-Katz test were purchased from Vestergaard-Frandsen, India. According to the manufacturers' instructions, the egg count recorded in each positive sample was multiplied by a factor of 24 to obtain the number of eggs per gram (epg) faeces. Intensity of infection was categorized using cut-off values recommended by WHO [9]. Accordingly, *A. lumbricoides, T. trichiura* and hookworm infections with egg counts less than 5000, 1000, and 2000 epg faeces respectively were categorized as light; those with egg counts of 5000–49999, 1000–9999, and 2000–3999 epg faeces respectively were categorized as moderately heavy; and those with egg counts of 50000 or over, 10000 or over, and 4000 epg faeces or over, respectively, were categorized as heavy infections. Faecal samples were examined at hospital/medical faculty laboratories located at a central, easily accessible point within each district (see Figure 1), in order to minimize sample transport time.

### Socio-economic data

Data regarding the school, the child's family and household sanitation were recorded in pre-tested questionnaires by trained, Tamil-speaking data collectors. The schools were also inspected for availability of water on tap in the school premises for flushing the toilet or for washing hands; availability of soap and water in the latrines; and cleanliness of the latrines. Adequacy in the number of toilets was calculated according to the standards adopted by the Education Ministry, which works out to roughly one latrine for every 50 children (Ministry of Education circular no 2007/21 of 8 October 2007, available at http://www.moe.gov.lk/web/images/stories/circulars/2007-21e.pdf).

A household latrine facility and usage score was constructed based on direct observation of the latrine by data collectors who were trained in this task. Their observations were recorded on a pre-tested form with categorical variables. This included the type of latrine used by the family (water-sealed/ pit/ bucket/ none), the availability of water on tap, soap, a door and a roof in the latrine, and the observation of faecal contamination in and around the latrine at time of inspection. Poor sanitation resulted in a low score (0 being the worst) while good sanitation (water-sealed latrine with roof and door, water on tap in latrine and soap available, no faecal contamination observed) was awarded a high score (maximum of 83). A cut-off of 73 was used for the logistic regression since this score divided the study population for which scores were available, into approximately equal halves. The score was not calculated for Nuwara Eliya district, as data collection was carried out by a different team, and records were not consistent.

### Geographic information

The geographical coordinates and altitude of every school was recorded using a hand held global positioning system (GPS) monitors (Trimble Juno SB from Trimble Navigation, Sunnyvale, CA USA; Garmin eTrex® H from Garmin International, Inc. Olathe, KS USA; or Magellan eXplorist 500 from Magellan Navigation Inc San Dimas, CA USA). Data recorded on GPS monitors were uploaded into a Geographic Information System (GIS) (ArcGIS, ESRI, Redlands, CA USA), and associated with attribute data on STH infection prevalence. A predicted prevalence map was created using ordinary kriging based on the prevalence of infection in schools. Kriging is a class of geo-statistical techniques used for optimal spatial prediction. They are statistically unbiased techniques (i.e., on average, the predicted value and the true value coincide) that minimize prediction mean-squared error, and provide a measure of uncertainty or variability in the predicted values. Kriging uses the semivariogram, a function of the distance and direction separating two locations, to quantify the spatial autocorrelation in the data. The semivariogram is then used to define the weights that determine the contribution of each data point to the prediction of new values at the unsampled locations. Ordinary kriging is a linear predictor, meaning that prediction at any location is obtained as a weighted average of neighbouring data. It assumes a constant but unknown mean, and estimates the mean value as a constant in the searching neighbourhood [10].

### Statistical analysis

Double entry was carried out using a database developed in EpiInfo Version 3.5.1 and data cleaned where there were discordant entries. Data analysis was done on SPSS Version 16.0 and R version 2.10. Multivariate analysis was carried out using logistic regression for each helminth infection, with the outcome variable categorized as presence or absence of infection. Taking into account the multistage sampling method used in the study design, the explanatory variables were grouped into district level (district, type of plantation and altitude), school level (duration since the last school medical inspection, total number of children in the school and availability of telephone at school), family level (mother's education beyond primary school, father's education beyond primary school, latrine score and family size) and individual level (sex, worm treatment within the preceding six months, use of footwear and condition of fingernails on inspection) variables. The assumptions for a logistic regression were checked with all the models.

Variables were added to the model in blocks by level in the following order: district, school, family and individual level. The significant variables in a block (p≤0.05) were retained in the model and the variables from the next lower level were added to the model. Continuous variables and categorical variables with more than two categories were dichotomized prior to being used as predictor variables. The selection of a model from among the models having the same or different number of explanatory variables was done based on the stability of model using the Akaike Information Criterion, degree of multi-co-linearity using variance inflation factor, significance of the odds ratio and relevance of the variable considering the existing knowledge. Father's education and mother's education had co-linearity and only mother's education was retained in the model considering its greater relevance to childcare.

## Results

**Figure 2:**
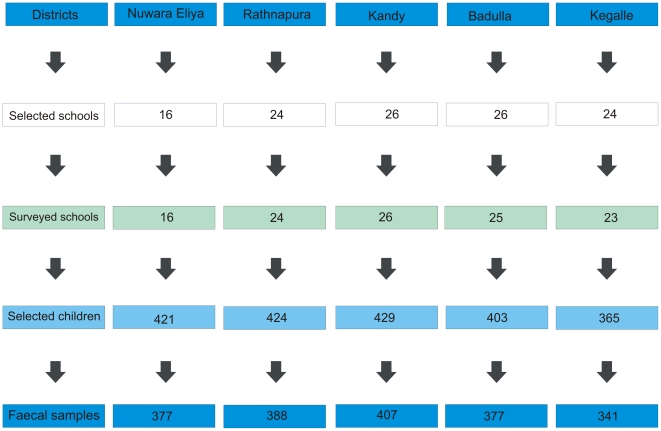
Flow chart showing numbers of selected schools, surveyed schools, selected children and faecal samples obtained in each district.

A total of 2,042 children in 114 schools in 5 districts (16 schools in Nuwara Eliya, 24 in Ratnapura, 26 in Kandy, 25 in Badulla and 23 in Kegalle, see Figures 1 and 2) were included in the survey. Two schools from the original list of selected schools (one in Badulla, and one in Kegalle) were subsequently excluded from the study because of difficulties in access.

The schools in Kegalle District were at the lowest mean altitude (270 m above sea level, SD 191 m); those in Ratnapura District were at a mean altitude of 339 m above sea level (SD 297 m); those in Kandy District at a mean altitude of 885 m above sea level (SD 172 m); those in Badulla District at a mean altitude of 1202 m above sea level (SD 228 m); while those in Nuwara Eliya District were at the highest mean elevation of 1454 m above sea level (SD 244 m).

### Soil-transmitted helminth infections

**Figure 3:**
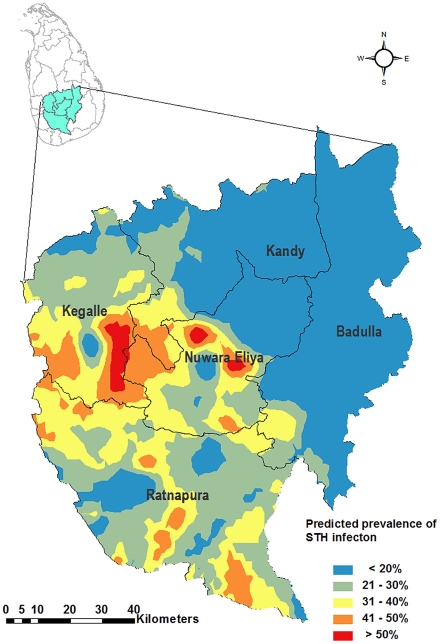
Predicted prevalence of infection with any one or more soil-transmitted helminth infection among children in the plantation sector in five districts of Sri Lanka.

Faecal samples were obtained from 1,890 children (compliance rate of 93%). Overall, 1072 children (52.5% of the study population) consisted of boys. Table 1 shows the prevalence of infection by district, while Figure 1 shows the proportion of children found infected in each study school. In four of the five districts surveyed, over 20% of the children were infected with one or more STH. The overall combined prevalence of infection was 29.0%. The commonest infection was *A. lumbricoides*, which was present in all five districts, as was *T. trichiura*. Hookworm infections (usually *Necator americanus* in Sri Lanka) were not detected in Nuwara Eliya and Badulla districts. Figure 3 presents a continuous surface map, which was created using ordinary kriging based on the prevalence of any STH infection at each school.

**Table 1:** Prevalence of soil-transmitted helminth infections in plantation sector school children by district.

District	Number examined	*Ascaris lumbricoides* eggs	*Trichuris trichiura* eggs	Hookworm eggs	Any STH egg
		Number positive (% infected)			
Nuwara Eliya	377	139 (36.9%)	15 (4.0%)	0	144 (38.2%)
Ratnapura	388	88 (22.7%)	24 (6.2%)	48 (12.4%)	122 (31.4%)
Kandy	407	87 (21.4%)	14 (3.4%)	7 (1.7%)	94 (23.1%)
Badulla	377	43 (11.4%)	7 (1.9%)	0	47 (12.5%)
Kegalle	341	105 (30.8%)	52 (15.3%)	34 (10.0%)	142 (41.6%)
**Total**	**1890**	**462 (24.4%)**	**112 (5.9%)**	**89 (4.7%)**	**549 (29.0%)**

Only 20/462 children (4.3%) with *A. lumbricoides* infection had infections of heavy intensity; 40.7% had moderately heavy infections; the rest (55.0%) had light infections. In contrast, nearly 90% of the *T. trichiura* infections were of light intensity and the remainder was of moderate intensity; no heavy *T. trichiura* infections were seen at all. In two of the three districts where hookworm was detected, all infections were of light intensity. In Ratnapura district only, a few heavy infections were seen (6.3% of infected children); while the large majority were light infections (40/48, 83.3%). Overall, as shown in Table 2, 219 children (11.6%) had moderate or heavy infections of one or more of the three infections.

**Table 2:** Proportion of infections of moderate – heavy intensity by district.

District	Number examined	*Ascaris lumbricoides*	*Trichuris trichiura*	Hookworm	Any STH
		Number with infections of moderate – heavy intensity (%)			
Nuwara Eliya	377	79 (21.0%)	1 (0.3%)	0	78 (21.0%)
Ratnapura	388	37 (9.5%)	4 (1.0%)	8 (2.1%)	42 (10.8%)
Kandy	407	36 (8.8%)	0	0	36 (8.8%)
Badulla	377	17 (4.5%)	0	0	17 (4.5%)
Kegalle	341	39 (11.4%)	7 (2.1%)	0	44 (13.2%)
**Total**	**1890**	**208 (11.0%)**	**12 (0.6%)**	**8 (0.4%)**	**211 (11.6%)**

### Personal hygiene

Overall, 22.4% of children (423/1884) were found to have overgrown, dirty fingernails on inspection during the survey. Among boys, this proportion was 25.5% (252/990), while amongst girls it was only 18.9% (166/877). Most of the children wore shoes to school (1417/1867, 75.9%), while another 21% (387/1867) wore slippers. Only a very small proportion of children (63/1867, 3.4%) had come barefoot to school. Boys were more likely to be barefoot than girls [46/991, (4.6%) vs 17/876, (1.9%) respectively]; whereas a higher proportion of girls were in shoes [685/876, (78.2%) vs 732/991 (73.9%) respectively] or slippers [174/876 (19.9%) vs 213/991 (21.5%) respectively] when compared with boys (Pearson chi-square  = 11.8, d.f. = 2, p = 0.003).

### De-worming and school medical inspections

On direct questioning, parents of 56.8% of children (1067/1880) claimed that the child had been de-wormed within the last 6 months, either through the school, or by the parents. The percentage of de-wormed children varied between districts: 74.5% (277/372) of children in Badulla had been de-wormed, whereas in Nuwara Eliya, only 46.7% (176/377) children had been de-wormed. The annual School Medical Inspection (SMI) had yet not been conducted in 27 of the 114 schools at the time of the survey. Since anthelmintics are usually administered during the School Medical Inspection, this indicated that the children in these 27 schools were unlikely to have received anthelmintics through school in the last 6 months.

### Sanitation and water supply in schools

The number of students per school latrine ranged from 6 to 209 with a mean of 66.9 students per latrine. Overall, only 61/113 schools (54.0%) met the Education Ministry norm regarding the number of latrines in relation to student population. When comparing districts, Kandy had the lowest numbers of students per school latrine (45.3) and 18/26 schools (69.2%) met the Education Ministry norm, whereas Nuwara Eliya had the highest number of students per school latrine (89.0) and only 25% of schools met the Education Ministry norm. Ratnapura and Badulla Districts had 79.7 and 62.2 students per school latrine, while 12/23 (52.2%) and 14/25 (56.0%) of schools respectively, met the Education Ministry norm. Almost all schools (98/114, 86.0%) had water on tap, either from a local water collection or from the public water supply. However, more than a third of schools (44/114, 38.6%) did not have water on tap in the toilets. Furthermore, just two schools in the Ratnapura district and one school in Nuwara Eliya district had soap available for hand-washing in the latrines; none of the other schools had soap for use by students.

### Household environment

Information was obtained on the educational attainment of 1516 fathers and 1469 mothers. Amongst the fathers, 11.9% had not attended school at all; this proportion was a little higher (15.2%) among mothers. Another 40.0% of fathers and 42.3% of mothers had attended only primary school.

With regard to the latrine score in the districts where household latrines were assessed, the score ranged from 0 to 83, with an overall mean of 64.8 (SD 21.3). There was no significant difference in mean scores between districts.

### Risk factors for infection

The results of the multi-variate analysis identified several risk factors for STH infection in this study population (see Table 3). At district level, altitude was a significant determinant of all three infections, with lower prevalence of infection at higher altitudes (>500 m above sea level). The effect was most marked for hookworm [Odds Ratio (OR) 0.08, 95% Confidence Interval (CI) 0.04–0.17], and least so for *A. lumbricoides* infection (OR 0.55, 95% CI 0.40–0.76). At school level, the duration since the last School Medical Inspection (>180 days) increased the risk of *A. lumbricoides* infection (OR 1.77, 95% CI 1.29–2.46) but not infection with either *T. trichiura* or hookworm. At household level, higher maternal school attainment (Grade 6 or higher) significantly reduced risk of all three infections (*A. lumbricoides* OR 0.40, 95% CI 0.28–0.57; *T. trichiura* OR 0.28, 95% CI 0.14–0.52; hookworm OR 0.47, 95% CI 0.25–0.84). Better household sanitation, as reflected by a latrine score of 74 or more, also significantly reduced the risk of *A. lumbricoides* (OR 0.70, 95% CI 0.51–0.97) and hookworm infection (OR 0.50, 95% CI 0.28–0.86), but not *T. trichiura*. At individual level, sex was a risk factor for hookworm, with girls being less at risk of infection than boys (OR 0.53, 95% CI 0.30–0.93). Although nail hygiene and use of footwear are generally considered protective factors, these variables were not identified as predictors in these models.

**Table 3:** Summary of multivariate analysis for risk of *A. lumbricoides*, *T. trichiura* and hookworm infection.

	*A. lumbricoides* infection (n = 462)	*T. trichiura* infection (n = 112)	Hookworm infection (n = 89)
Explanatory variable*	Odds ratio (95% confidence interval)		
Altitude [More than 500 m]	0.55 (0.40-0.76) [n = 263]	0.26 (0.15-0.44) [n = 41]	0.08 (0.04-0.17) [n = 11]
Duration since last SMI [180 days or more]	1.77 (1.29-2.46) [n = 193]	1.00 (1.00-1.00) [n = 43]	0.93 (0.54-1.63) [n = 43]
Mother's education [grade 6 or more]	0.40 (0.28-0.57) [n = 107]	0.28 (0.14-0.52) [n = 18]	0.47 (0.25-0.84) [n = 20]
Latrine score [74 or more]	0.70 (0.51-0.97) [n = 125]	1.00 (0.99-1.01) [n = 46]	0.50 (0.28-0.86) [n = 33]
Sex [Female]	0.76 (0.55-1.05) [n = 199]	0.91 (0.54-1.52) [n = 51]	0.53 (0.30-0.93) [n = 32]

*: maximum variance inflation factor was 1.02.

Numbers within square brackets indicate the number of infected children positive for the given explanatory variable.

## Discussion

These results suggest that STH infections are still highly prevalent among schoolchildren in the plantation sector. At 29.0%, the overall prevalence of infection is well above the level at which the WHO recommends introduction of annual mass de-worming. A significant proportion of infected children (11.6%) had infections of moderate or heavy intensity, indicating that they are at particular risk of morbidity. It is likely that observed prevalence rates are an underestimate of true prevalence, since only a single faecal sample was examined, using only the Kato-Katz technique for detection of eggs. Use of serial faecal samples and multiple diagnostic techniques have been reported to increase detection rates, especially with regard to hookworm and light infections [11], [12]. Extremely high prevalence rates, such as those observed in the plantation sector of Sri Lanka in the 1990s, are necessarily accompanied by very high levels of environmental contamination with the eggs and larvae of STH, consequent on pollution of the soil with human faeces. Under favorable conditions, these stages remain viable in soil for many months, sometimes years, and so re-infection after anthelmintic treatment is virtually inevitable, and prevalence rates rebound rapidly [2]. For example, in a study conducted on school children in Pemba Island, Tanzania, when prevalence was well over 90%, infection intensities reached pre-treatment levels by 6 months after treatment with single dose albendazole or mebendazole [13].

Two recent studies from Zanzibar, Tanzania, which also had helminth prevalence rates >90% among school children in the 1990s, found that despite implementation of mass deworming for about 15 years, overall helminth prevalence (which included infection with *A. lumbricoides*, hookworm, *T. trichiura*, *Strongyloides stercoralis* and *Schistosoma haematobium*) remained high. A cross-sectional study was carried out in 2 schools in Unguja Island, Zanzibar, about 6 months after the last school-based anthelmintic treatment was carried out, and compared with data obtained from the same schools in 1994 [14]. Overall prevalence of STH infection had dropped significantly from 98.9% in 1994, but was still high at 59.7%. Another study that compared prevalence in one rural and one peri-urban community in Unguja Island found overall helminth prevalences of 73.7% and 48.9% respectively [15].

It is possible that the greater decline in prevalence seen in the Sri Lankan plantation sector over a similar period of about 15 years, starting from similar prevalences of over 90%, may be attributed to the fact that the mass deworming programme was biannual in Sri Lanka, thus reducing rebound in infection between treatment rounds. Concomitant efforts to improve sanitary facilities in the estates is also likely to have contributed to the less than expected increase in the prevalence of STH infections following cessation of the mass deworming programme.

China has also seen a significant decline in the prevalence of soil-transmitted helminth infections, between its first national survey conducted in 1990 and the second in 2003, with the standardized rates of hookworm, *A. lumbricoides* and *T. trichiura* infections declining by 60.7%, 71.3% and 73.6% respectively [16]. This decline has been attributed to the conduct of mass deworming programmes targeting school children in many parts of China, along with the introduction of appropriate health education in schools, improvements in water supplies and sanitation, and the increasing use of chemical fertilizer instead of night soil in agriculture. The Ministry of Health has now adopted a three-pronged approach in its National Control Programme on Important Parasitic Diseases for 2006–2015. They include large scale deworming with benzimidazoles; providing clean water and adequate sanitation; and health education programmes [17].

The Republic of Korea also had a major problem with STH infections, with an overall prevalence of 84.3% in 1971. Nationwide mass de-worming targeting schoolchildren was conducted twice a year from 1969 to 1995, and successive quinquennial nationwide surveys showed prevalence to decline gradually, reaching a nadir of 2.4% in 1997 [18]. However, the Korean national economy also grew very rapidly during this period, and living standards improved remarkably, along with sanitation and agricultural technology. These factors would have undoubtedly contributed to the reduction in STH prevalence.

Since it is apparent that mass deworming must be resumed in the plantation sector of Sri Lanka, the question arises as to how this should be done. There are two ways by which anthelmintics could be delivered to children in the plantation sector. One would be the adoption of a mass de-worming day, when all children in plantation sector school are given anthelmintics on a single, designated day, preceded by adequate preparation in the form of teacher training and advance publicity to raise awareness regarding the issue. This is the usual practice in many countries where school-based de-worming is carried out on a national scale [2]. The other approach would be to strengthen the existing School Medical Inspection programme, where a team from the nearest Medical Officer of Health office visits the school on an annual basis. Traditionally, de-worming of schoolchildren has been a part of the SMI programme. However, de-worming may not occur if the inspection is not conducted for some reason or sufficient stocks of anthelmintics are not available when the SMI is conducted. Also, while all children are examined during the SMI in schools where the total student population is <200, in the larger schools, only children in Grades 1, 4 and 7 are inspected and treated. This policy will need to be changed, so that all children in all plantation sector schools are treated regardless of school size.

The choice of anthelmintic to be used in mass treatment needs to be given some consideration. At present, single dose mebendazole (500 mg) tablets are given to children during the SMI. However, our results suggest that a history of recent anthelmintic treatment significantly reduced only the risk of infection with *A. lumbricoides*, and had no long-term impact on *T. trichiura* or hookworm infections. This is not surprising given that single dose mebendazole is known to be much less effective against hookworm and *T. trichiura* than against *A. lumbricoides* [19]. It may be advisable to consider the possibility of using either single-dose albendazole or a 3-day course of mebendazole, particularly in Ratnapura and Kegalle Districts, where *T. trichiura* and hookworm infections show a relatively high prevalence. It should be noted here that although mebendazole 500 mg was used in much of the mass deworming program in the plantation sector, when a survey carried out 2 years after commencement of the program showed only a very modest decrease in hookworm infections, mebendazole was replaced with albendazole 400 mg in the low altitude plantations where hookworm was most prevalent [20]. A re-evaluation carried out two years after the introduction of albendazole showed that the prevalence of hookworm had reduced sharply on the three plantations that were surveyed [20].

Our findings also strongly suggest that de-worming alone will not eliminate the problem of soil-transmitted helminth infections. After the sector-wide survey conducted in 1994 showed extremely high prevalence rates, biannual mass de-worming of school aged children was carried out for nearly 10 years before its cessation in 2005. During this period, however, other measures that could also reduce transmission, such as improved sanitation and health education, were not greatly emphasized. Given that transmission of all three infections is dependent upon contamination of soil with human faeces, good sanitation plays an extremely important role in breaking the cycle of transmission and preventing infection, as evidenced by identification of the household latrine score as a significant risk factor. Household sanitation is one of the areas in which the plantation sector in Sri Lanka lags behind the rest of the country. It has been estimated that only 85.1% of plantation sector households had sustainable access to improved sanitation in 2006/07, whereas in the rural and urban sectors, this proportion was 94.8% and 91.5% respectively [1]. Thus in order to sustain gains achieved by the re-introduction of preventive chemotherapy, other strategies such as information education and communication strategies, behavior change interventions and community led total sanitation projects will need to be pursued more vigorously.

Poor maternal education was also identified as a risk factor for infection, as has been found in other studies [21]-[23]. Given that 15.2% of mothers in this study had not attended school at all, and 42.3% had only completed primary school, this is not surprising. Unfortunately this is part of a vicious cycle where poverty and general lack of education among parents, and mothers in particular, increases the risk of worm infections among children, which in turn has a negative impact on the children's cognitive ability [2]. Poor performance and achievement levels in school results in lack of job opportunities and low income in adult life and so the cycle continues.

We also found that the risk of infection with any of the three soil-transmitted helminth infections decreased with increasing altitude. This association was most marked in relation to hookworm, and least evident with *A. lumbricoides* infections. It is known that free-living infective stages in the environment develop and die at temperature-dependent rates [24]. It is likely that larval survival declines at higher altitudes, where ambient temperatures are much lower than at lower altitudes.

Badulla District had the lowest observed prevalence rates among the five districts surveyed. It is possible that this may be partly due to altitude (since it had schools with the second highest mean altitude), but it could also be due to children being given anthelmintics by their parents, which was observed to be highest (74.5%) in Badulla. The mean years of school attended by both fathers and mothers were also highest in Badulla (6.34 and 6.04 years respectively), but household latrine scores, sanitation in schools, and SMI coverage was not significantly different from other districts. Thus it is possible that the low prevalence in this district is maintained by the high frequency of deworming by parents.

In conclusion, the results of this study indicate that there is a need to resume mass de-worming on an annual basis in the plantation sector in Sri Lanka, along with stronger emphasis on other long-term control measures such as improved sanitation and water supplies, and better health education programmes. The results highlight the fact that even after 10 years of biannual mass chemotherapy, when the initial force of transmission is strong and living conditions remain poor, the prevalence of soil-transmitted infections can bounce back after cessation of mass deworming.
